# Validation of exercise-response genes in skeletal muscle cells of Thoroughbred racing horses

**DOI:** 10.5713/ajas.18.0749

**Published:** 2019-02-14

**Authors:** Doh Hoon Kim, Hyo Gun Lee, Nipin Sp, Dong Young Kang, Kyoung-Jin Jang, Hak Kyo Lee, Byung-Wook Cho, Young Mok Yang

**Affiliations:** 1 Department of Pathology, School of Medicine, Institute of Biomedical Science and Technology, Konkuk University, Chungju 27478, Korea; 2 Department of Animal Science, College of Natural Resources and Life Sciences, Pusan National University, Miryang 50463, Korea; 3 Department of Animal Biotechnology, Chonbuk National University, Jeonju 54896, Korea; 4 Life and Industry Convergence Research Institute, Pusan National University, Miryang 50463, Korea

**Keywords:** Thoroughbred, Exercise Stress, Skeletal Muscle Cells, Gene Expression

## Abstract

**Objective:**

To understand the athletic characteristics of Thoroughbreds, high-throughput analysis has been conducted using horse muscle tissue. However, an *in vitro* system has been lacking for studying and validating genes from in silico data. The aim of this study is to validate genes from differentially expressed genes (DEGs) of our previous RNA-sequencing data *in vitro*. Also, we investigated the effects of exercise-induced stress including heat, oxidative, hypoxic and cortisol stress on horse skeletal muscle derived cells with the top six upregulated genes of DEGs.

**Methods:**

Enriched pathway analysis was conducted using the Database for Annotation, Visualization, and Integrated Discovery (DAVID) tool with upregulated genes in horse skeletal muscle tissue after exercise. Among the candidates, the top six genes were analysed through geneMANIA to investigate gene networks. Muscle cells derived from neonatal horse skeletal tissue were maintained and subjected to exercise-related stressors. Transcriptional changes in the top six genes followed by stressors were investigated using quantitative reverse transcription-polymerase chain reaction (qRT-PCR).

**Results:**

The inflammation response pathway was the most commonly upregulated pathway after horse exercise. Under non-cytotoxic conditions of exercise-related stressors, the transcriptional response of the top six genes was different among types of stress. Oxidative stress yielded the most similar expression pattern to DEGs.

**Conclusion:**

Our results indicate that transcriptional change after horse exercise in skeletal muscle tissue strongly relates to stress response. The qRT-PCR results showed that stressors contribute differently to the transcriptional regulation. These results would be valuable information to understand horse exercise in the stress aspect.

## INTRODUCTION

The development of athletic performance in Thoroughbreds is impacted by several factors such as genetics, environment and training. For this purpose, a new molecular biology approach is needed for a deeper understanding and improvement of the horse athletic performances. Therefore, ongoing research is mainly focused on the factors and genes related to better performances [1,2]. However, even in the case of a horse with excellent traits, excessive exercise causes a variety of stress on the muscle tissue, which has a significant effect on post-exercise performance. For instance, rapid cell respiration from intense exercise has been reported to induce not only hypoxia but also oxidative stress and heat stress of the muscle tissue [3–5]. In addition, to maintain homeostasis during horse exercise, a large amount of cortisol is secreted from the pituitary gland [6], and this hormone affects the muscle tissue. These stressors lead to change in gene expressions related to energy metabolism, inflammation, cell proliferation, and protein metabolism. Therefore, it is necessary to study not only the genetic factors for speed but also stresses induced by exercise.

With completion of the horse genome project, many genes have been identified and databases were built. The next-generation sequencing (NGS) method yields important clues for developing molecular markers and identifying overall transcriptional changes of muscle tissue after exercise. In a first RNA-sequencing analysis for horse exercise, the authors confirmed changes in transcripts with equine exercise and proposed that a panel of genes changed are related to the adaptive processes to exercise, muscle growth, and metabolic processes [7]. In 2012, Park et al [8] also performed RNA-sequencing using skeletal muscle tissue after Thoroughbred exercise, producing a list of candidate genes related to horse racing performance. Another analysis of the changes in whole transcriptome profiling of blood during exercise was conducted in 2013 [9]. These authors focused on exercise-induced stress and suggested that several groups of differentially expressed genes are involved in inflammation and the immune response. However, animal experiments using horses have high costs and ethical issues, along with difficulties in understanding the effects of individual stresses because of the complexity of diverse stressors. In addition, because muscle tissue is composed of various immune cells, blood vessel, nerve cells and blood cells, it is difficult to directly study the mechanism of stress response using muscle tissue. Therefore, the development of an *in vitro* system to validate the key genes of NGS data is required.

The present study aims to develop an *in vitro* system for validating the major genes previously identified using NGS [10]. In addition, we analyzed the transcriptional changes in major genes of NGS data in exercise-induced stress conditions including heat, oxidative, hypoxic, and cortisol stress.

## MATERIALS AND METHODS

### Primary horse muscle cell culture

The Pusan National University-Institutional Animal Care and Use Committee approved the study design (Approval Number: PNU-2015-0864). A skeletal muscle tissue biopsy was performed on the leg of a neonatal Thoroughbred to culture horse muscle cells (HMCs). HMCs were cultured according to the previous study [11]. Briefly, primary HMCs were maintained and sub-passaged in Medium 199 (Gibco, Grand Island, NY, USA) supplemented with 10% foetal bovine serum (Invitrogen, Carlsbad, CA, USA) and 1% antibiotic-antimycotic (Gibco, USA). HMCs were incubated in a humidified atmosphere with 5% CO_2_ at 37°C. Routine fluid renewals were made three times a week. At 70% to 80% confluence, cells were gently washed twice with phosphate-buffered saline and harvested using 0.05% trypsin-ethylenediaminetetraacetic acid (Welgene, Daegu, Korea) to isolate total RNA. We chose 42°C for heat stress according to the physiological condition of horse skeletal muscle tissue after exercise [12]. A value of 2% of O_2_ was determined to induce the hypoxic condition according to the previous work using a hypoxic chamber [13].

### RNA extraction and cDNA synthesis

Total RNA was extracted from HMCs by RNA isolation reagent (TRIzol, Invitrogen, Karlsruhe, Germany). Next, 200 μL of chloroform was added to remove cells from the organic solvent, then the mixture was shaken for 10 s, maintained at 4°C for 5 min, and then centrifuged at 4°C for 15 min. The supernatant was removed and added to a new test tube, mixed with an equal amount of isopropanol, and maintained at 4°C for 15 min to collect RNA pellets. Isopropanol was removed from the solution by centrifuging at 4°C for 15 min, then was sterilized with 75% ethanol and dissolved in RNase-free water. The purity of the extracted RNA was confirmed by measuring absorbance at 230 nm and 260 nm using a spectrophotometer (Nanodrop Technologies Inc., Wilmington, DE, USA), and only RNA with a purity (optical density value of 230 nm/260 nm) greater than 1.8 was selected and stored at −70°C for further experiments. To synthesize cDNA, 1 μg of RNA, 1 μL of oligo-dT (Invitrogen, Waltham, MA, USA) and RNase-free water were added. The RNA was denatured at 80°C for 3 min, and cDNA was synthesized using 4 μL of 5× reverse transcription (RT) buffer, 5 μL of 2 mM dNTPs, 0.5 μL of RNase inhibitor and 1 μL of M-MLV (Moloney-murine leukaemia virus) RT (Promega, Madison, WI, USA).

### Real time-quantitative polymerase chain reaction

NCBI (http://www.ncbi.nlm.nih.gov) and the Ensembl Genome Browser (www.ensembl.org) were used to retrieve gene sequence information. The primers for amplification of the genes (Table 1) were designed using PRIMER3 software (http://bioinfo.ut.ee/primer3-0.4.0/). Real-time quantitative polymerase chain reaction (qPCR) was performed using a thermal cycler (C1000 Thermal Cycler, Bio Rad, Hercules, CA, USA) to measure the relevant expression of target genes in 25 μL of reaction solution, constructed as follows: 2 μL diluted cDNA (50 ng/μL) added to 14 μL SYBR green master mix (Bio Rad, USA) and 1 μL each of 5 pmol/μL diluted forward and reverse primers. The conditions used for the real-time qPCR were as follows: initial denaturation at 94°C for 10 min followed by 40 cycles of denaturation at 94°C for 10 s, annealing at 60°C for 10 s, and extension at 72°C for 30 s. All measurements were carried out in triplicate, and the 2^−ΔΔCt^ method was used to determine relative gene expression. The relative expression of target genes was normalized to glyceraldehyde-3-phospate dehydrogenase.

### MTT assay

Cell viability was assayed by measuring blue formazan that was metabolized from MTT by mitochondrial dehydrogenase. The HMCs were re-suspended in the medium one day before H_2_O_2_ and cortisol treatment, at a density of 2×10^5^ cells per well in 24-well culture plates. Liquid medium was replaced with fresh medium containing dimethyl sulfoxide (DMSO) for control. The HMCs were incubated with various concentrations of H_2_O_2_ (Junsei, Tokyo, Japan) and cortisol (Merck, Darmstadt, Germany). MTT (5 mg/mL) was added to each well and incubated for 4 h at 37°C. The formazan product formed was dissolved by adding 200 μL DMSO to each well, and the absorbance was measured at 570 nm on a microplate reader (Ultra Multifunctional Microplate Reader, Tecan US Inc., Durham, NC, USA). All measurements were performed in triplicate and repeated at least three times.

### Morphological analysis

The HMCs were plated on 60-mm culture dishes at a concentration of 5×10^5^ cells/plate and incubated at 37°C under 5% CO_2_ for 4 h under each stressor. Then, cell images were acquired by using an inverted microscope.

### Bioinformatics analysis

The DEG data from the previous research was used for bioinformatics analysis [10]. As all genes were listed in Ensembl gene IDs, the horse Ensemble gene IDs were converted to official gene symbols by cross-matching with human Ensembl gene IDs and the official gene symbols. The official gene symbols of human homologues of equine genes were used for functional clustering and enrichment analyzes using the Database for Annotation, Visualization, and Integrated Discovery (DAVID) tool. The functional categories based on co-occurrence with a set of upregulated genes in DEGs were analyzed using the Kyoto encyclopedia of genes and genomes (KEGG) pathway tool in the DAVID. The statistical significance of pathway over-representation was calculated based on the DAVID software EASE score (p value based on a modified Fisher’s exact t-test) and corrected for multiple testing using the Benjamini-Hochberg stepdown correction.

Gene network analysis using GeneMANIA (https://genemania.org) was conducted with the top six genes from the DEG data. Database information including co-expression, co-localization, shared protein domains, and predicted functional relationships between genes were collected from GeneMANIA.

### Statistical analysis

All data for qRT-PCR were expressed as mean±standard deviation from three independent experiments. GraphPad Prism software (GraphPad Software, La Jolla, CA, USA) was used to evaluate the data. The statistical significance (* p< 0.05, ** p<0.01, or *** p<0.001) was assessed by two sample student’s *t*-test.

## RESULTS

### Stress-related pathways and gene networks of the top six upregulated genes after exercise

**Figure 1:**
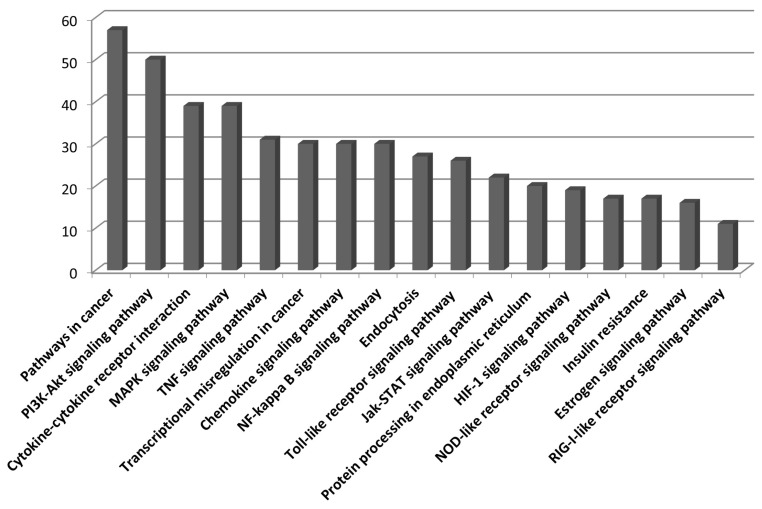
KEGG pathway categories of upregulated genes in horse skeletal muscle tissue after exercise. 1,186 genes sorted from upregulated transcripts after horse exercise were used to analyze KEGG pathway annotation. The number of genes in each category is shown along the Y-axis while the different KEGG pathways are shown along the X-axis. KEGG, Kyoto encyclopedia of genes and genomes.

**Figure 2:**
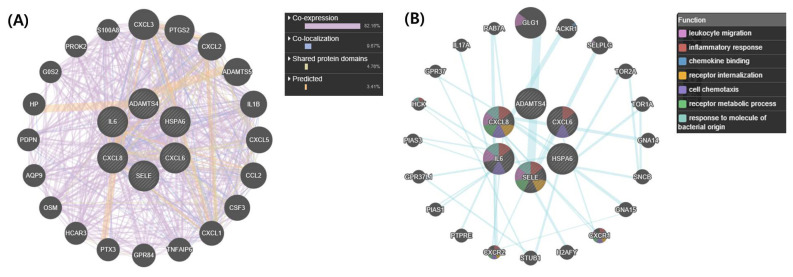
Gene network analysis of all interactions of the top six genes in upregulated differentially expressed genes. (A) Gene network of interactions with co-expression, co-localization, shared protein domains, and predicted functional relationships. (B) Related pathways of the top six genes. The colours in each gene indicate the related function of the pathway. *IL6*, *CXCL8* (*IL8*), *CXCL6*, *ADAMTS4*, *HSPA6*, and *SELE* are shown in inner circles while related genes are shown in outer circles. *IL6*, interleukin 6; *CXCL8*, interleukin 8; *CXCL6*, chemokine (C-X-C motif) ligand 6; *ADAMTS4*, A disintegrin and metalloproteinase with thrombospondin motifs 4; *HSPA6*, heat shock 70 kDa protein 6; *SELE*, selectin E.

We used the upregulated DEGs data derived from our previous study [10]. We cut off the list based on a false discovery rate (FDR) of <0.01. Then, a set of genes was sorted depending on Log2fold change (Log_2_FC) value (Log_2_FC>1). Finally, we obtained 1,383 DEGs that included 1,186 known and 197 unknown upregulated genes. To analyze pathways and gene networks that relate to the list of upregulated genes, we used the DAVID bioinformatics tool with the known genes. The 1,186 genes upregulated after horse exercise in skeletal muscle tissue were involved in the multiple biological pathways derived from the database of the KEGG pathway (Figure 1). The pathways were related to inflammatory responses such as cytokine–cytokine receptor interaction, MAPK signalling pathway, tumor necrosis factor signalling pathway, chemokine signalling pathway, NF-kappa B signalling pathway, toll-like receptor signalling pathway, and Nucleotide-Binding Oligomerization Domain-like receptor pathway. Among the 1,186 known upregulated genes, 17 genes (1.4%) were significantly upregulated after exercise (Log_2_FC>7). To deepen the gene networks, we selected the top six upregulated genes: interleukin 6 (*IL6*), *IL8*, chemokine (C-X-C motif) ligand 6 (*CXCL6*), A disintegrin and metalloproteinase with thrombospondin motifs 4 (*ADAMTS4*), heat shock 70 kDa protein 6 (*HSPA6*), and selectin E (*SELE*) (Table 2). We then performed GeneMANIA analysis to determine the relationships among the six genes. The network consisted of 26 genes, including the six selected genes and 20 additional genes identified by GeneMANIA (Figure 2A). Co-expression (82.16%), co-localization (9.67%), shared protein domains (4.76%), and predicted functional relationships (3.41%) were confirmed by literature searching. The pathway result consisted of 26 genes, including six selected genes and 20 additional genes (Figure 2B). The common pathways of DAVID and GeneMANIA were related to an inflammatory response such as cytokine–cytokine receptor interaction and the chemokine signalling pathway. These results indicate that the upregulated genes after horse exercise in skeletal muscle tissue strongly relate to the stress response.

### Exercise-related stresses in racing horse skeletal muscle cells

**Figure 3:**
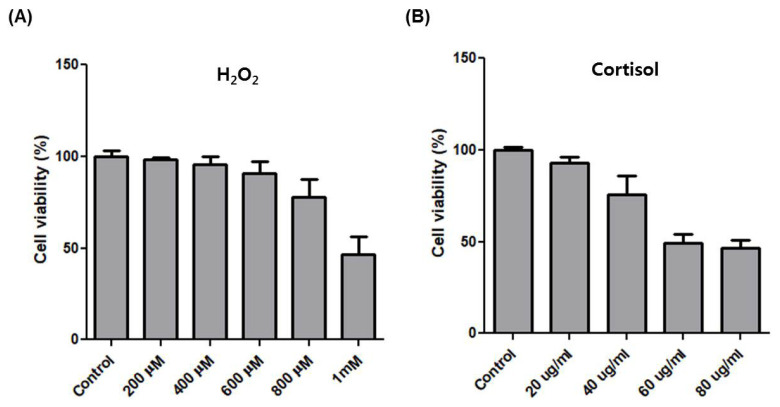
The effect of H_2_O_2_ and cortisol on cell viability of HMCs. (A) MTT assay to measure cell viability in HMCs after treatment with H_2_O_2_ at different concentrations for 4 h. (B) MTT assay to measure cell viability in HMCs after treatment with cortisol at different concentrations for 4 h. HMCs, horse muscle cells.

After horse exercise, the horse skeletal muscle tissue is subjected to various stresses. We selected the representative exercise stressors including heat, oxidative, hypoxic, and hormone stress. Then, we established an *in vitro* system to validate the effect of each stressor on the transcription of the top six upregulated genes. We selected H_2_O_2_ and cortisol to apply oxidative and hormone stress, respectively. The effect of oxidative stress with H_2_O_2_ at 200 μM to 1 mM on cell viability of HMCs was assessed by the MTT assay. H_2_O_2_ at 800 μM reduced cell viability by approximately 75% compared to control (Figure 3A). When hormone stress with cortisol at 20 μg/mL to 80 μg/mL was applied, cortisol at 40 μg/mL reduced cell viability by approximately 75% compared to control (Figure 3B). We applied 600 μM H_2_O_2_ and 20 μg/mL cortisol to choose a concentration at which the cell viability was unaffected. Next, 42°C and 2% O_2_ were applied for heat and hypoxic stress, respectively.

**Figure 4:**
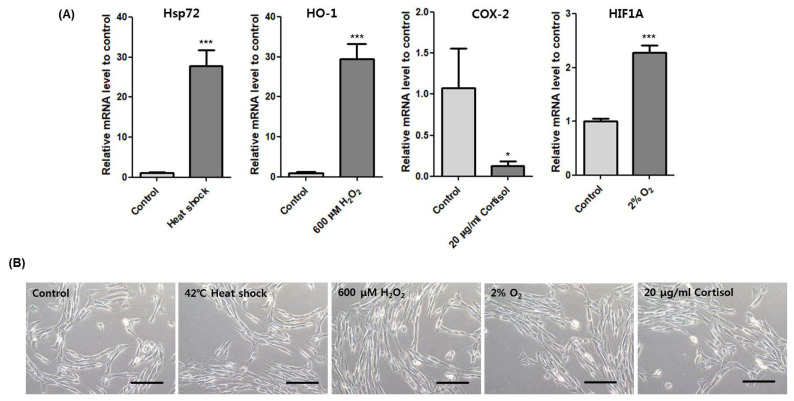
Stress marker expression under morphologically unaffected state. (A) Relative expression of *Hsp72* after incubation at 42°C for 1 h. Relative expression of *HO-1* after treatment of 600 μM H_2_O_2_ for 4 h. Relative expression of *COX-2* after treatment of 20 μg/mL cortisol for 4 h. Relative expression of *HIF1A* after incubation at 2% O_2_ for 4 h. Asterisk indicates differences that are statistically significant (* p<0.05, ** p<0.01, *** p<0.001). (B) Cell morphology of HMCs after incubation at each stress condition. Scale bar: 10 μm. HMCs, horse muscle cells; *Hsp72*, heat shock 70 kDa protein 1; *HO-1*, heme oxygenase-1; *COX-2*, cyclooxygenase-2; *HIF1A*, hypoxia-inducible factor 1-alpha.

Then, we used several marker genes to confirm the induction of each stressor including heat shock 70 kDa protein 1 (*Hsp72*), heme oxygenase-1 (*HO-1*), cyclooxygenase-2 (*COX-2*), and hypoxia-inducible factor 1-alpha (*HIF1A*) for heat, oxidative, cortisol, and hypoxic stress, respectively [14–17]. Transcription of *Hsp72*, *HO-1*, and *HIF1A* increased significantly and *COX-2* decreased (Figure 4A). Moreover, cell morphologies of each stress treatment were similar to control (Figure 4B). These results show that heat, oxidative, hypoxic and cortisol stress were induced under non-lethal conditions.

### Effects of exercise-related stress on the transcription of the top six upregulated genes

**Figure 5:**
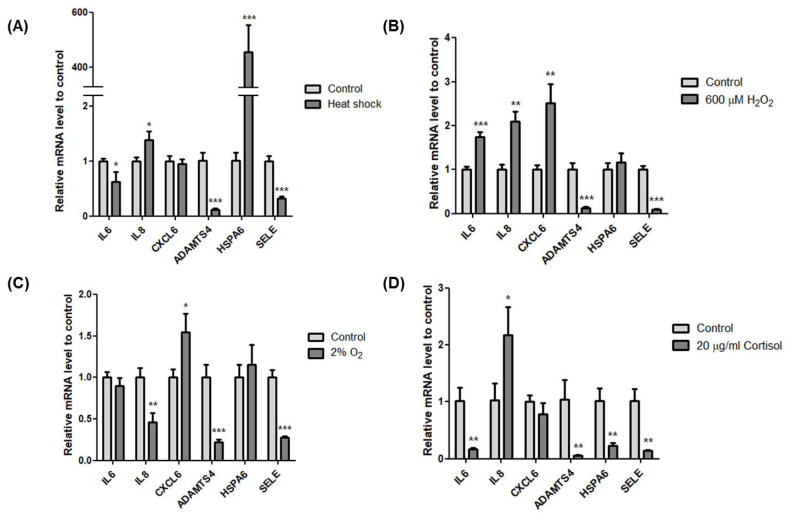
The effect of exercise-related stressors on the expression of the top six genes. (A) Relative expression of the six genes using cDNA of HMCs after incubation at 42°C for 1 h. (B) Relative expression of the six genes using cDNA of HMCs after treatment with 600 μM H_2_O_2_ for 4 h. (C) Relative expression of the six genes using cDNA of HMCs after incubation at 2% O_2_ for 4 h. (D) Relative expression of the six genes using cDNA of HMCs after treatment with 20 μg/mL cortisol for 4 h. Glyceraldehyde-3-phospate dehydrogenase (*GAPDH*) was used as the reference gene. All experiments were conducted independently in triplicate. Asterisk indicates differences that are statistically significant (* p<0.05, ** p<0.01, *** p<0.001). HMCs, horse muscle cells; *IL6*, Interleukin 6; *IL8*, Interleukin 8; *CXCL6*, Chemokine (C-X-C motif) ligand 6; *ADAMTS4*, A disintegrin and metalloproteinase with thrombospondin motifs 4; *HSPA6*, Heat shock 70 kDa protein 6; *SELE*, selectin E.

Next, we validated the effect of the exercise-related stressors on the transcriptional induction of *IL6*, *IL8*, *CXCL6*, *ADAMTS4*, *HSPA6*, and *SELE*. *IL8* (* p<0.05) and *HSP6A* (*** p<0.001) increased while *IL6* (* p<0.05), *ADAMTS4* (*** p<0.001), and *SELE* (*** p<0.001) decreased after heat stress (Figure 5A). Interestingly, *HSP6A* (*** p<0.001) increased significantly, almost 400 times more than control. In oxidative stress with 600 μM of H_2_O_2_, *IL6* (*** p<0.001), *IL8* (** p<0.01), and *CXCL6* (** p<0.01) significantly increased while *ADAMTS4* (*** p< 0.001) and *SELE* (*** p<0.001) significantly decreased (Figure 5B). Hypoxic condition with 2% O_2_ increased *CXCL6* (* p< 0.05). However, the other genes were downregulated except *HSPA6*, which showed a slight increase (Figure 5C). Cortisol stress with 20 μg/mL showed decreased expression of all six genes except *IL8* (* p<0.05) (Figure 5D). Although all six genes were upregulated after horse exercise *in silico*, these data clearly indicate that heat, oxidative, hypoxic, and hormone stress contribute differently to the transcriptional regulation of the top six upregulated genes. Also, oxidative stress led to expression similar to that seen *in silico*.

## DISCUSSION

The RNA-seq method using horse muscle tissue is an efficient approach for screening genes related to horse exercise [8]. Especially, information from DEGs can be used to narrow down the putative genes potentially associated with exercise. However, horse exercise generates a variety of stresses in skeletal muscle tissue. Indeed, various stress-related signalling pathways are involved in horse exercise (Figures 1, 2), so it is important to validate the impact of stressors on genes from RNA-seq data after horse exercise.

Even though the six genes were upregulated after exercise stress [10], the effects of the four stress conditions used in this study on these genes differ in transcriptional regulation (Figure 5). Among the exercise-related stressors, oxidative stress showed the most similar expression pattern with DEG data except for *ADAMTS4* and *SELE* (Figure 5B). Interestingly, all conditions used in this study induced downregulation of *ADAMTS4* and *SELE* in HMCs. Extracellular matrix is an important part of the structure and function of muscle and not only constitutes a framework for the attachment of contractile cells but also has a role in cell proliferation, differentiation, migration, and polarization [18]. ADAMTS4 is a secreted protease that can degrade various proteoglycans to regulate remodelling of extracellular matrix and is expressed in skeletal muscle [19]. Similar to horse exercise RNA-seq data [10], acute exercise enhanced *ADAMTS4* expression by more than 18 fold in human skeletal muscle [20]. However, no conditions used in this study could induce upregulation of *ADMATS4* expression. The transcriptional discrepancy between DEGs and validation traces to differences in tissue and cells or additional exercise-related stress. SELE plays an important role in recruiting leukocytes to the injury site during inflammation. Regarding exercise, no significant changes in serum concentration of circulating E-selectin have been observed after exercise [21]. However, most studies of SELE have been conducted in vascular endothelial cells and leukocytes, not in muscle cells. Therefore, further investigation is needed to confirm the SELE function in muscle cells.

The temperature of horse muscle increases 43.3°C±0.7°C at the end of exercise [12]. When we exposed HMCs to 42°C, expression of the heat stress marker *HSPA1A* (*Hsp72*) significantly increased. In this condition, the expression of *HSP6A* dramatically increased more than 400 fold (Figure 5A). HSP6A is an Hsp70 chaperone that is inducible in response to stress. HSP6A shows no or low expression levels under non-stressed conditions [22,23], but its expression is important to cell survival under thermal stress or toxic agents. A knockdown study of both *HSPA1A* and *HSP6A* shows that HSPA1A is the primary responder and that HSP6A is a secondary responder under proteotoxic stress [24]. HSP6A can form a complex with HSPA1A and work cooperatively following stress [25]. Furthermore, a new heat shock element in the promoter region of *HSP6A* was defined, suggesting that an HSF-like factor mediates thermal stress in human keratinocytes [26]. Indeed, *HSPA6* increases at 24 h after resistance exercise in human skeletal muscle [27]. In line with previous studies, our results show that among the top six genes, *HSPA6* is the most inducible in response to heat stress and that heat stress in muscle is the main cause of *HSPA6* upregulation after exercise.

Skeletal muscle is a secretory organ of cytokines, which are involved in inflammatory processes. Exercise provokes an increase in a subset of cytokines in muscle, called myokines. IL6 and IL8 are well studied among the myokines. Contraction of skeletal muscle results in releasing of IL6, and plasma levels of IL6 increase with exercise [28]. IL6 activates AMPK and PI3-kinase to increase glucose uptake in skeletal muscle [29]. Indeed, horse exercise induces upregulation of genes related to the PI3K-Akt signalling pathway (Figure 1), indicating a requirement for glucose homeostasis. Our result implies that not only physical contracting of skeletal muscle but also oxidative stress contributes to increased *IL6* expression (Figure 5B). Similarly, *IL8* levels in plasma increase in response to eccentric exercise [30]. IL8 is a known chemokine that attracts primarily neutrophils and also serves as an angiogenic factor. Although the role of IL8 in skeletal muscle needs to be clarified, our results indicate that heat, oxidative, and cortisol stressors contribute to induction of *IL8* expression in skeletal muscle (Figures 5A, 5B, 5D).

Finally, the discrepancy between DEG data and our study in terms of the extent of expression may be attributable to other factors related to exercise or synergetic or offset effects of stressors used in this work. Nevertheless, this study is the first to validate genes from DEG data in cells derived from horse muscle tissue. These results will be helpful for investigating and validating the effect of exercise-induced stresses. Furthermore, horse skeletal muscle cells could be used as an *in vitro* system for developing anti-stress substances and drugs to reduce exercise-related stress.

## Figures and Tables

**Table 1:** Primer information used in this study

Target gene	Sequence (5′ to 3′)	Annealing Tm (°C)	Product size (bp)
*IL6*	Forward: CACCACTGGTCTTTCGGAGT		
	Reverse: TCAGGGGTGGTTACTTCTGG	60	156
*IL8*	Forward: GCTTTCTGCAGCTCTGTGTG		
	Reverse: TCTGAGTTTTCGCAGTGTGG	60	153
*CXCL6*	Forward: AGAGAACTGCGTTGCATGTG		
	Reverse: GTTTTTCAATGCGTGGTCCT	61	242
*ADAMTS4*	Forward: TGCATCTGCCTGTGACTTTC		
	Reverse: CCAGGGTGAATGTTTGGTCT	61	173
*HSPA6*	Forward: CGTGAGGCTGAGCAGTACAA		
	Reverse: CCAGTTCCCTCTTCTGATGC	61	107
*SELE*	Forward: TTCCGGAAGTTTCCAAAGTG		
	Reverse: AAGCCTTCCTCACAGCTGAA	61	243
*HO-1*	Forward: GGGTGATCGAAGAGGTCAAA		
	Reverse: GCCACCAGAAAGCTGAGTGT	60	244
*Hsp72*	Forward: CGACCTCAACAAGAGCATCA		
	Reverse: AAGATCTGCGTCTGCTTGGT	60	213
*HIF1A*	Forward: CACCACTGGTCTTTCGGAGT		
	Reverse: TCAGGGGTGGTTACTTCTGG	60	156
*COX-2*	Forward: AACAGGAGCATCCAGAATGG		
	Reverse: AAAAGCAGCTCTGGGTCAAA	60	151
*GAPDH*	Forward: GGTGAAGGTCGGAGTAAACG		
	Reverse: AATGAAGGGGTCATTGATGG	60	106

*IL6*, interleukin 6; CXCL6, Chemokine (C-X-C motif) ligand 6; *ADAMTS4*, A disintegrin and metalloproteinase with thrombospondin motifs 4; *HSPA6*, heat shock 70 kDa protein 6; *SELE*, selectin E; *HO-1*, heme oxygenase-1; *Hsp72*, heat shock 70 kDa protein 1; *HIF1A*, hypoxia-inducible factor 1-alpha; *COX-2*, cyclooxygenase-2; *GAPDH*, glyceraldehyde-3-phospate dehydrogenase.

**Table 2:** List of genes validated in this study

Ensembl gene ID	Gene symbol (Full name)	Log_2_FC	p-value	FDR
ENSECAG00000016482	*IL6* (Interleukin 6)	12.69	1.21E-186	7.17E-183
ENSECAG00000015342	*IL8* (Interleukin 8)	12.30	1.92E-130	3.80E-127
ENSECAG00000012742	*CXCL6* (Chemokine (C-X-C motif) ligand 6)	10.21	1.35E-72	3.34E-70
ENSECAG00000024172	*ADAMTS4* (A disintegrin and metalloproteinase with thrombospondin motifs 4)	10.00	5.75E-195	6.81E-191
ENSECAG00000004180	*HSPA6* (Heat shock 70 kDa protein 6)	8.52	1.22E-140	3.62E-137
ENSECAG00000008423	*SELE* (Selectin E)	8.19	5.97E-122	7.86E-119

FC, fold change; FDR, false discovery rate.
